# Supplementary material for: Atomic-resolution imaging of electrically induced oxygen vacancy migration and phase transformation in SrCoO2.5-σ
Source: Nat Commun. 2017 Jul 24;8:104. doi: 10.1038/s41467-017-00121-6 (PMC5524633; doi:10.1038/s41467-017-00121-6)
Supplement: Supplementary file 1 — Supplementary Information [file 41467_2017_121_MOESM1_ESM.pdf]

File name: Supplementary Information
Description: Supplementary Figures and Supplementary Table 1

File name: Peer Review File
Description:

a

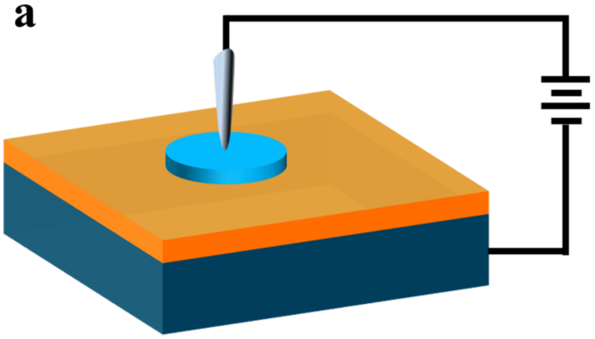

b

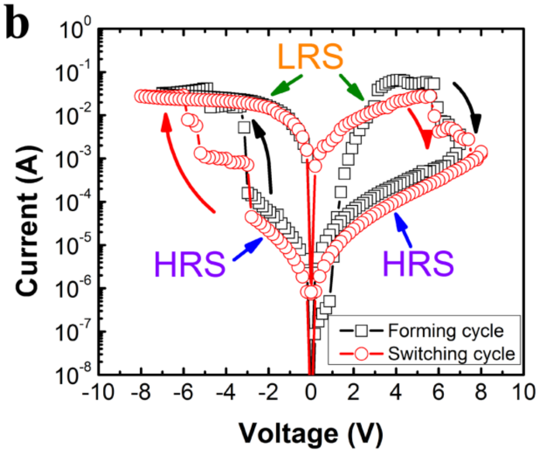

Supplementary Figure 1. Forming and switching cycles outside a TEM. (**a**) Configuration schematics of thin-film resistance switching outside a TEM. Device structure of Pt/$SrCoO_{2.5}$/$SrTiO_3$(Nb) for resistance switching composes of a top electrode (the Pt electrode (blue) with a disc radius of 120 *u*m) and a bottom electrode (highly conductive Nb-doped $SrTiO_3$ substrate (dark blue)). The film thickness is ~ 50 nm. (**b**) Current-Voltage (I-V) curves in forming and switching cycles with the configuration descripted in (**a**). During the forming cycle as indicated by the dark squares and arrows, the film transformed from the pristine state into a high resistance state (HRS) at ~7 V and into a low-resistance state (LRS) by a reverse bias of ~(-3 V). The resistance can be switched reversibly and steadily as displayed by the red circles and arrows.

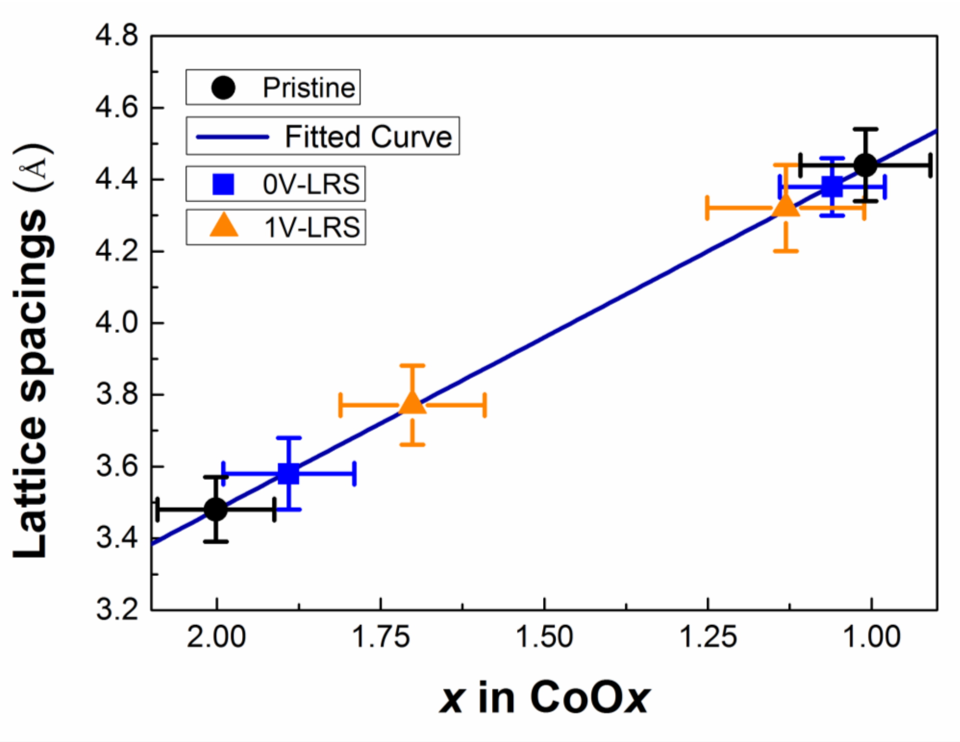

Supplementary Figure 2. Estimation of oxygen occupancy. Here we used pristine $SrCoO_{2.5}$, repeated unit of which can be expressed as [SrO-CoO1-SrO-CoO2], as the reference to extract the linear coefficient of the expression. That is, x=1 for the d_{SrT} (4.44±0.10 Å) containing CoO layer; x=2 for d_{SrO} (3.48±0.09 Å) containing CoO2 layer as shown in Supplementary Table 1. The error bars is the standard error calculated in OriginPro, obtained from five HAADF images in each phase. Assuming a linear relationship between the oxygen content (x) and lattice spacing (d), we obtained that

$$x=5.625-1.0416*d$$

Thus, we can infer the x in CoOx by measuring the interplannar spacings d_{SrT} and d_{SrO}.

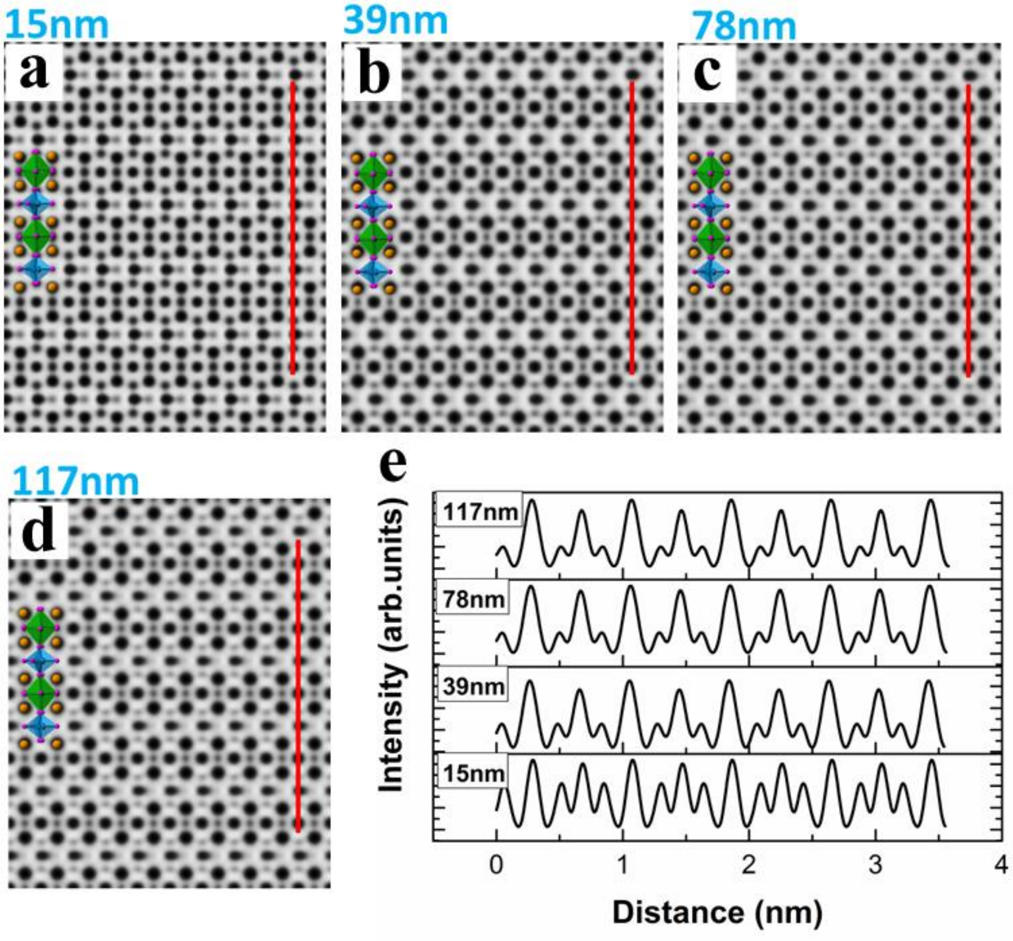
15nm
a
39nm
b
78nm
c
117nm
d
e
117nm
78nm
39nm
15nm
Intensity (arb.units)
0
1
2
3
4
Distance (nm)

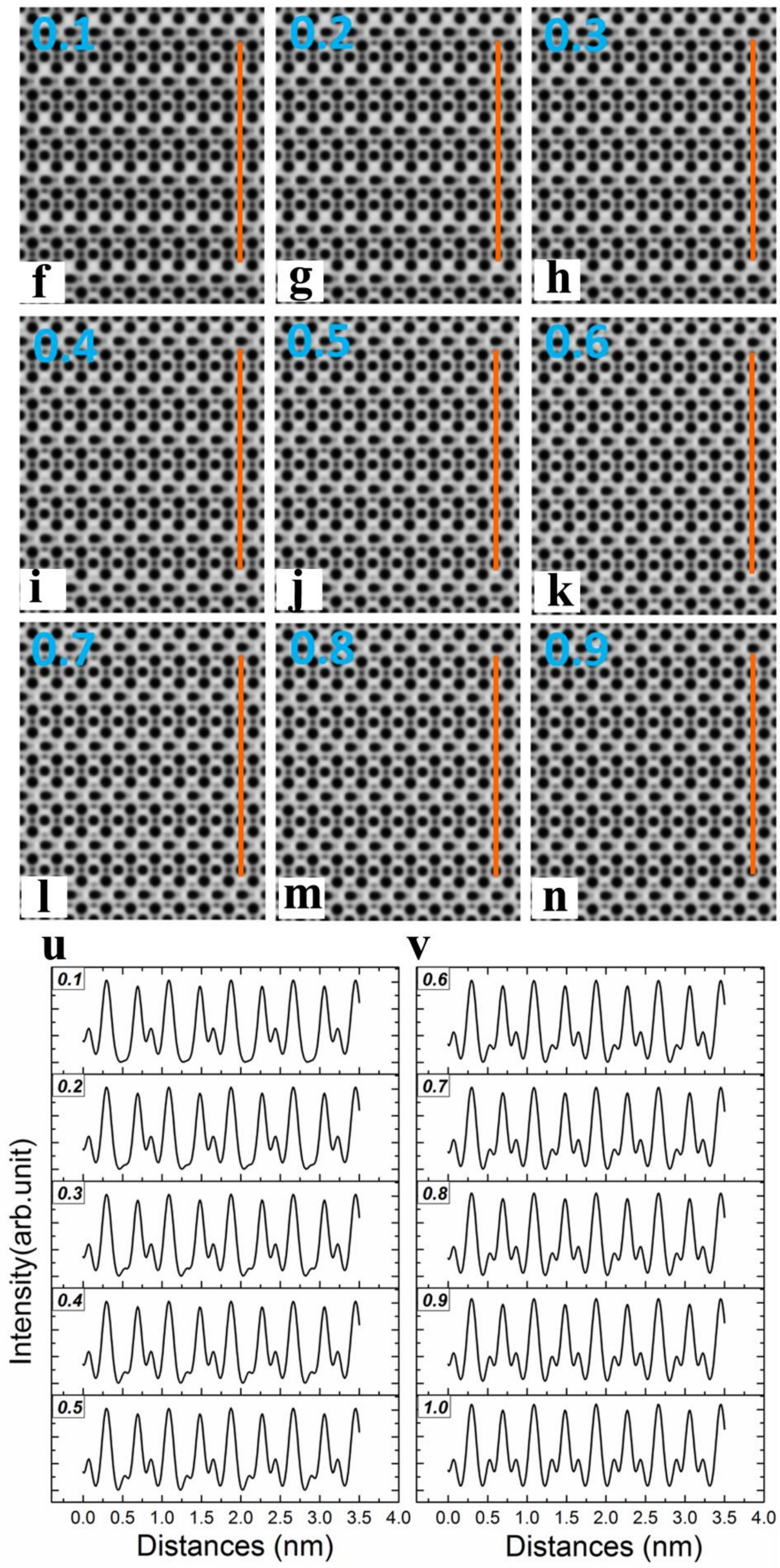

0.1
0.2
0.3
f
g
h
0.4
0.5
0.6
i
j
k
0.7
0.8
0.9
l
m
n
u
v
Intensity(arb.unit)
0.1
0.2
0.3
0.4
0.5
0.6
0.7
0.8
0.9
1.0
0.0 0.5 1.0 1.5 2.0 2.5 3.0 3.5 4.0
Distances (nm)
0.0 0.5 1.0 1.5 2.0 2.5 3.0 3.5 4.0
Distances (nm)

Supplementary Figure 3. Simulated ABF images. (a-e) Simulations on pristine $SrCoO_{2.5}$ phase at different thickness. We can find that the 100 slices is the most appropriate thickness by comprehensively comparing the relative contrast of Co and O columns. In addition, the lamella milled by the Ga-ion beam is around 30-50nm, which also supports the thickness of 39nm used in the simulations. **(f-n)** Simulated ABF images corresponding to different occupations of O_A at Sr2O layers with a thickness of 39nm. The occupation of oxygen in every second SrO layer varied from 0.1 to 1. Simulation condition: thickness: 39nm; collection angle for HAADF image: 90-250 mrad; collection angle for the ABF image: 11-22mrad. Probe size: 0.78nm. Defocus: 30 angstrom. **(u-v)** Line profiles of inversed ABF contrast as indicated by the red lines in **(b)**.

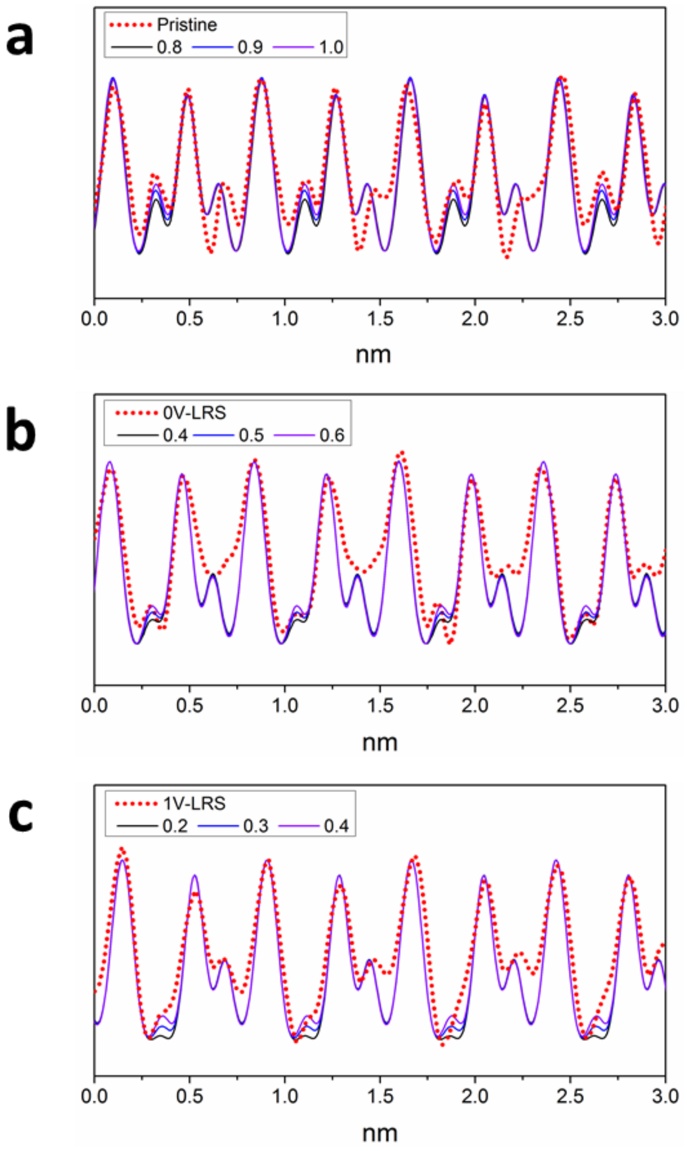

Supplementary Figure 4. Comparison of ABF contrast. We estimated an approximate range for the oxygen occupancy in the SrO planes by the systematic comparisons. For example, we compared the ABF image of 0V-LRS with the simulations on the oxygen occupancy of 0.4, 0.5 and 0.6, we can find that the case of 0.5 and 0.6 can match the decreased contrast in sites indicated by the green and blue arrows, respectively. Considering the fluctuation of the image contrast between 0.5 and 0.6 cases, the error bar of 0.1 is given. Thus, the y is averagely estimated as 0.55±0.1. All the results with error estimation can be found in the revised Supplementary Table 1.

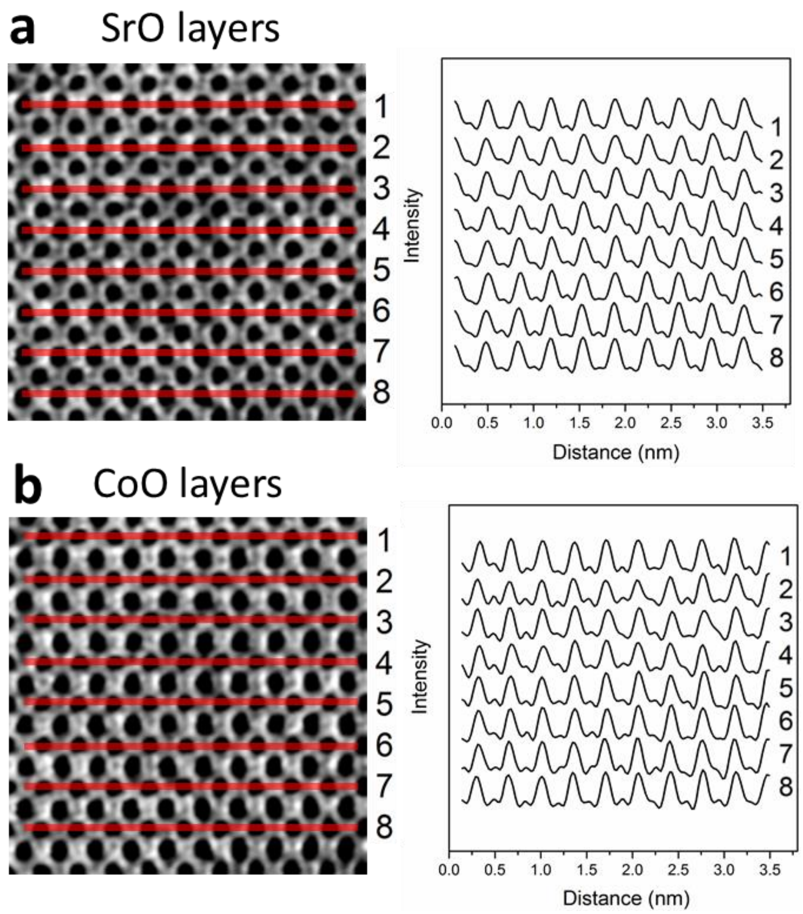

Supplementary Figure 5. Line profiles of inversed ABF contrast of SrO and CoO layers. Red lines indicated positions on the ABF images with corresponding numbers. It demonstrates the homogenous contrast of oxygen columns for SrO, as well as CoO layers, except some fluctuations.

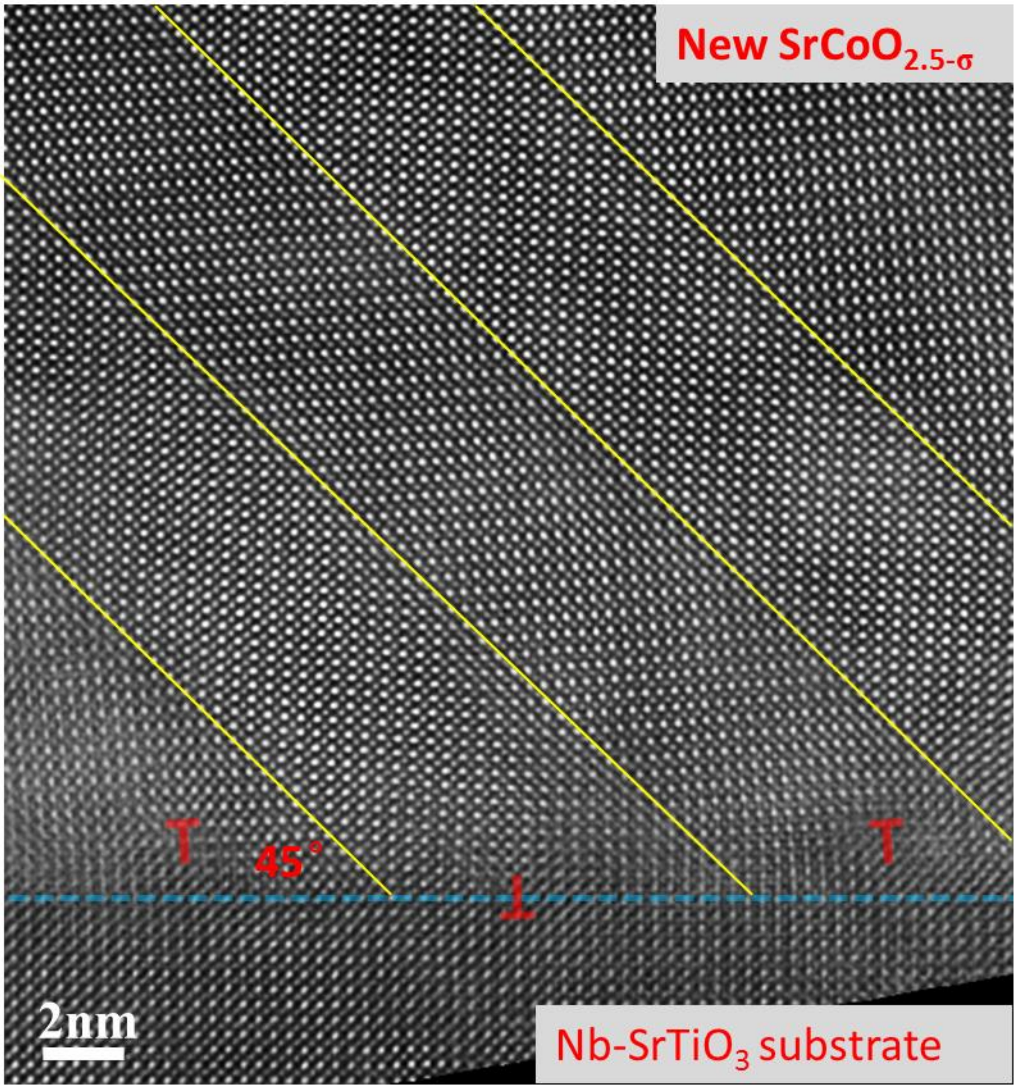

Supplementary Figure 6. HAADF image of new $SrCoO_{2.5-\sigma}$. Twinned lamellae orientates along the direction with an intersection angle of ~45° to the $[001]_p$ of the Nb-doped $SrTiO_3$ substrate. The edge dislocations near the interface are indicated by the red "T", which accommodate the lattice mismatch between the twinned domains and the Nb-doped $SrTiO_3$ substrate.

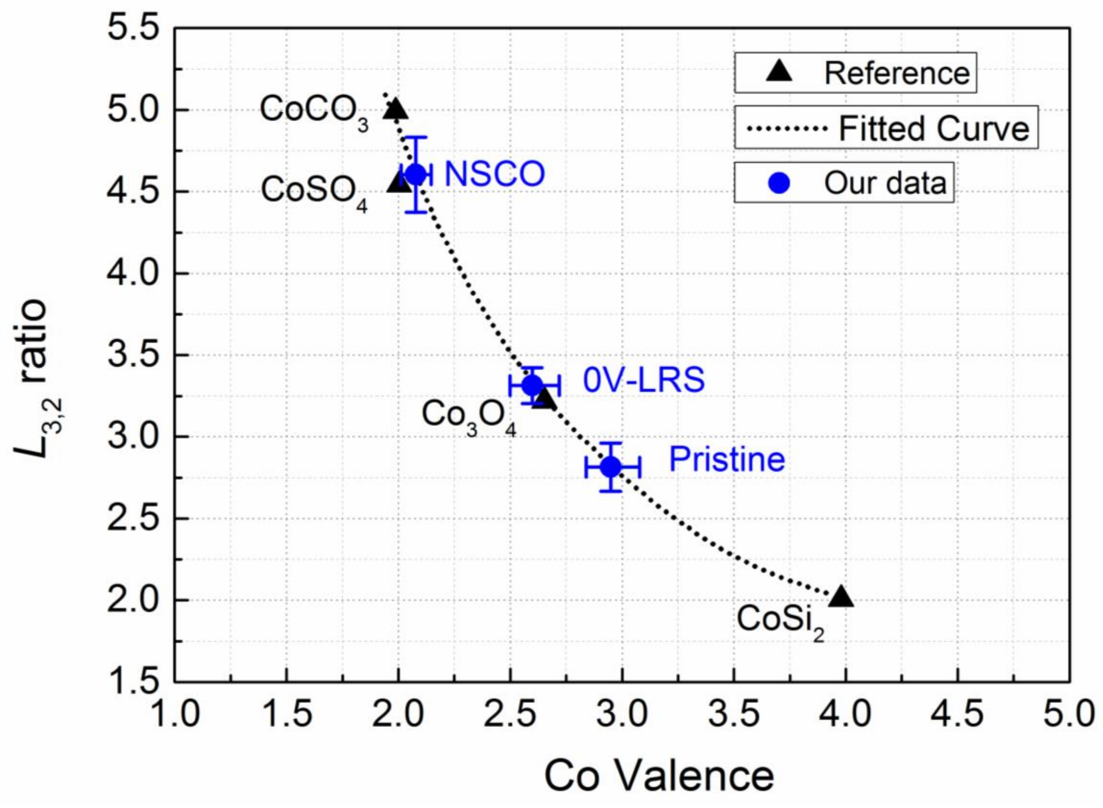

Supplementary Figure 7. Comparative plot to estimate the valence state of Co. The dotted line is the empirical fitting curve of Co valence state as a function of Co L_3/L_2 intensity ratio with reference to the known standard samples (solid triangles) established by Wang et al. [Micron 31 (2000) 571–580]. The ratio values of 2.82±0.15, 3.32±0.11, 4.61±0.23 had been obtained for the pristine, 0V-LRS and the NSCO phase from more than ten spectra in each sample, respectively. As shown in Supplementary Figure 5, we plotted our data on the empirical fitting curve to estimate valence state of Co in three states, which correspond to 2.84~3.08, 2.54~2.67 and 2.02~2.15 for the pristine, 0V-LRS and the NSCO phase, respectively.

Accordingly, we can also induce the oxygen content from the valence states of Co due to the electroneutrality principle. We obtained the oxygen content: 2.42~2.54, 2.27~2.34 and 2.01~2.07 for the pristine, 0V-LRS and the NSCO phase, respectively, which is in good agreement with the results from the HAADF and ABF images, where the 2.50±0.10, 2.26±0.14, 2.09±0.16 obtained for the pristine, 0V-LRS and 1V-LRS states.

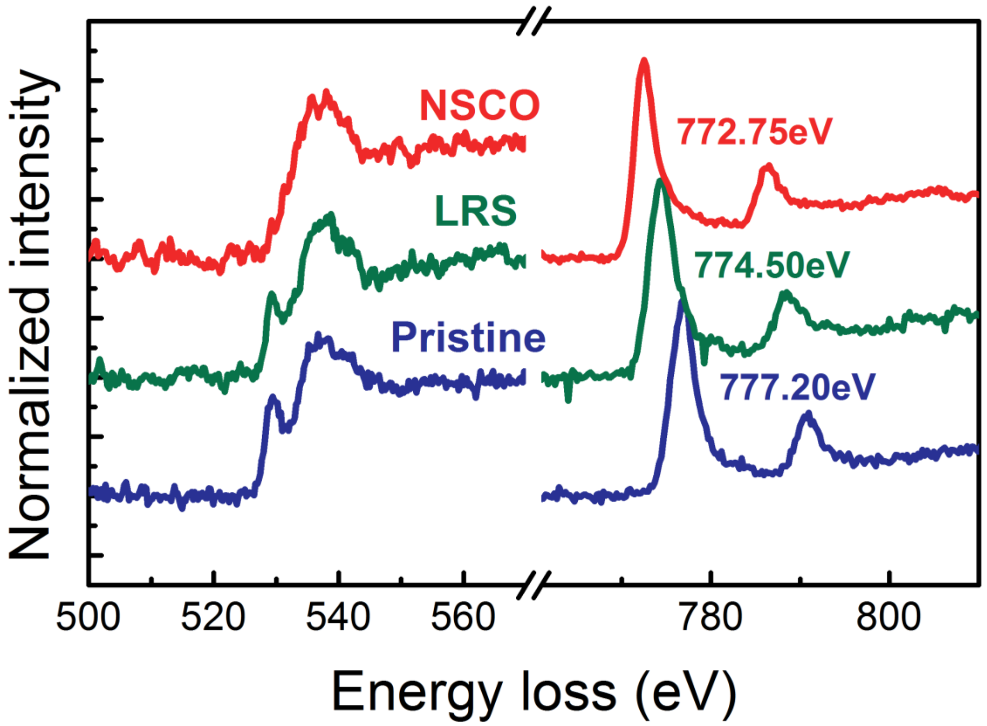

Supplementary Figure 8. Electron energy-loss spectra for three states with offset. The blue dotted line labels the pre-peak of the O K edges. The Co L_3 edge shift towards a lower energy of 774.5 eV and 772.7 eV in the LRS and NSCO (see in Supplementary Figure 6), respectively, indicating the valence decrease of Co^{3+} and an obvious change in electronic structure of the NSCO phase.

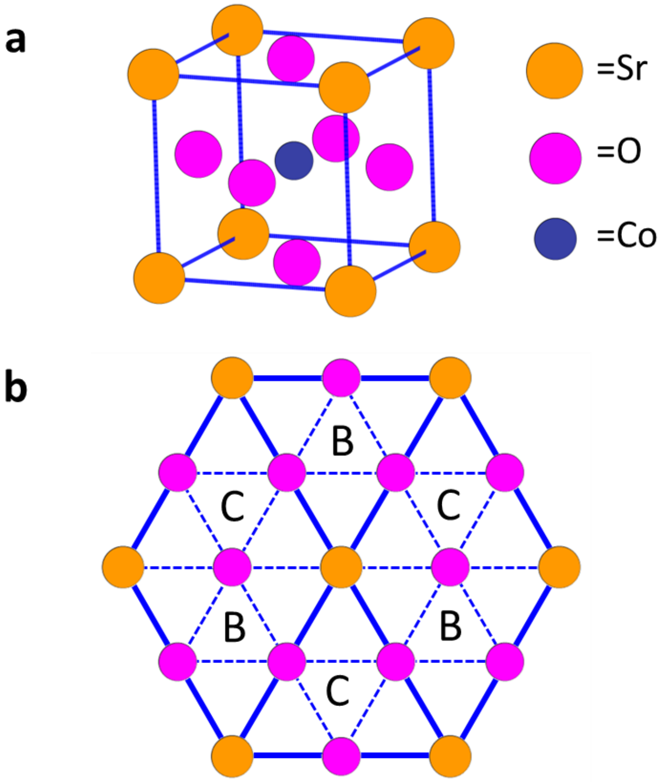

Supplementary Figure 9. Schematic layer model of ideal cubic $SrCoO_3$. (**a**) Hypothetical perovskite $SrCoO_3$. SrO_3 in an L12 (Cu_3Au) ordered arrangement on an *fcc* lattice with Co in the central octahedral void. (**b**) Perovskite as stacking of close-packed (111) planes. The plane shown is the plane of A-sites in the …ABC… stacking of close-packed planes that generates the *fcc* structure. The sites labeled B and C in the figure show the positions of the Sr atoms in the planes of B- and C-sites that are sequentially stacked on the A-plane to create the perovskite structure shown in (**a**).

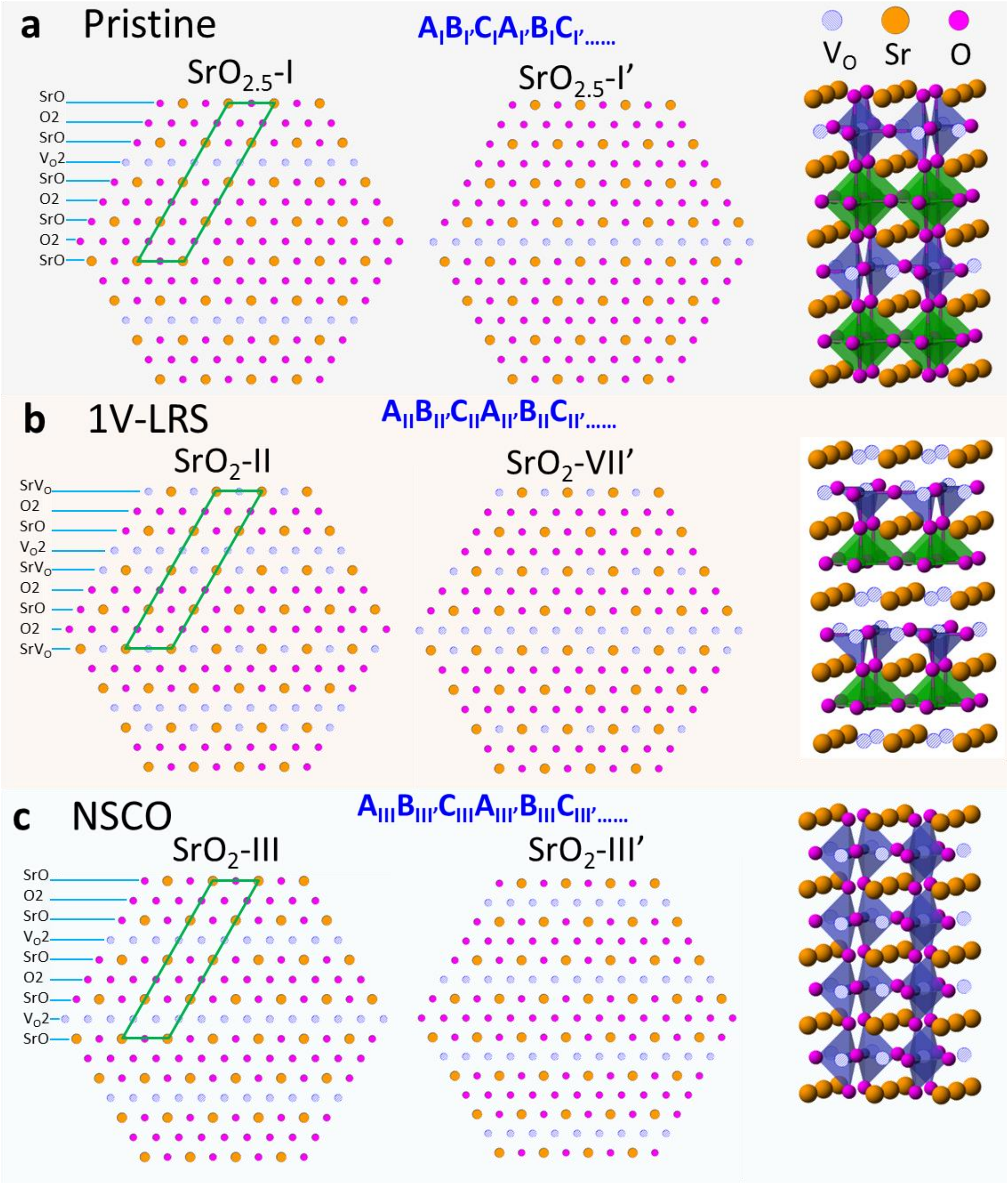

Supplementary Figure 10. Schematics of SrO_{3-x} (111) layer models of three phases. (**a**) $SrO_{2.5}$-I (111) layer of pristine $SrCoO_{2.5}$ with a stacking unit of [SrO-O2-SrO-V_O2-SrO-O2-SrO-O2-SrO] and $SrO_{2.5}$-I' (111) layer with an atomic displacement. The ABCABC…stacking of two (111) layers (for example $SrO_{2.5}$-I and $SrO_{2.5}$-I'), that is [$A_IB_{I'}C_IA_{I'}B_IC_{I'}$ ……], produces the oxygen-deficient pervoskite structure ($SrCoO_{2.5}$). (**b**) SrO_2-II (111) layer of 1V-LRS with a stacking unit of

[SrV$_O$-O2-SrO-V$_O$2-SrV$_O$-O2-SrO-O2-SrV$_O$] and $SrO_{2.5}$-II' (111) layer with an atomic displacement. (**c**) SrO_2-III (111) layer of NSCO with a stacking unit of [SrO-O2-SrO-V$_O$2-SrO-V$_O$2-SrO-O2-SrO] and $SrO_{2.5}$-III' (111) layer with an atomic displacement. For clarity, we ignore the lattice relaxation near the vacant sites in the models.

	Pristine	0V-LRS	1V-LRS
**d_{SrT}(Å)**	4.44±0.10	4.38±0.08	4.32±0.12
**d_{SrO}(Å)**	3.48±0.09	3.58±0.10	3.77±0.11
x1	1±0.10	1.06±0.08	1.13±0.12
x2	2±0.09	1.89±0.10	1.70±0.11
y* in every second SrO*y	0.95±0.10	0.55±0.10	0.35±0.10
**Averaged *x***	1.50±0.10	1.48±0.09	1.41±0.11
**Averaged *y***	0.95±0.10	0.78±0.05	0.68±0.05
**$SrCoO_{2.5-\sigma}$**	2.45±0.10	2.26±0.14	2.09±0.16

Supplementary Table 1. Estimation of oxygen occupation. The change of the oxygen stoichiometry can be estimated by summing *x* and *y* for the CoO*x* and SrO*y* layers, where *x* is estimated by the linear relation with out-of-plane Sr-Sr distances $d_{Sr\text{-}Sr}$. Here we used pristine $SrCoO_{2.5}$, repeated unit of which can be expressed as [SrO-CoO1-SrO-CoO2], as the reference to extract the linear coefficient of the expression. That is, *x*=1 for the d_{SrT} (4.44±0.10 Å) containing CoO layer; *x*=2 for d_{SrO} (3.48±0.09 Å) containing CoO2 layer as shown in Supplementray Table S1. Assuming a linear relationship between the oxygen content (*x*) and lattice spacing (d), we obtained that

$$x=5.625-1.0416*d$$

Thus, we can infer the *x* in CoO*x* by measuring the interplannar spacings d_{SrT} and d_{SrO}.

y is obtained by comparing the ABF contrast in experimental and simulation images as shown in Fig. 2i-k and Supplementary Figure 4, respectively.
